# Risk factors for acute unplanned tracheostomy during panendoscopy in HNSCC patients

**DOI:** 10.1371/journal.pone.0207171

**Published:** 2018-12-05

**Authors:** Friederike Eissner, Georg Haymerle, Markus Brunner

**Affiliations:** Department of Otorhinolaryngology, Head and Neck Surgery, Medical University of Vienna, Vienna, Austria; Public Library of Science, UNITED KINGDOM

## Abstract

**Background:**

Despite of careful pre-operative risk evaluation some patients require an acute unplanned tracheostomy during panendoscopy.

**Methods:**

Risk factors of patients requiring an unplanned tracheostomy during panendoscopy (n = 32) were compared to a control group with panendoscopy without tracheostomy (n = 180).

**Results:**

2131 panendoscopies for Head and Neck squamous cell carcinoma were performed at our Department between 2000 and 2014. Unplanned tracheostomies were necessary in 1.6% of all panendoscopies. Patients with laryngeal cancer (*p* = 0.001) or abnormal activated partial thromboplastin time (aPTT) (*p* = 0.03) had a statistically significant higher risk of unplanned tracheostomy. Regression analysis showed that patients with advanced laryngeal cancer had an almost 6 times higher risk for tracheostomy than patients with early stage oropharyngeal cancer.

**Conclusions:**

We identified abnormal aPTT and laryngeal carcinoma as significant predictors for unplanned tracheostomy during panendoscopy. The results of our study could improve preoperative risk evaluation in HNSCC patients.

## Introduction

Head and neck squamous cell carcinoma (HNSCC) is the sixth most commonly diagnosed cancer worldwide, accounting for about 6% of all new cancers and 5.2% of all cancer specific deaths [1]. All HNSCC patients in Austria undergo panendoscopy to confirm the histologic diagnosis, to exclude a second primary, to determine the tumour extent and to evaluate potential surgical therapy options before beginning the treatment [2–5]. Before every operation, surgeons and anaesthesiologists evaluate all patients regarding their airway management. Due to a lack of scientific data this risk evaluation is based on clinical experience. Currently there are several studies suggesting scoring systems to predict the risk of unplanned tracheostomy in head and neck surgery although non of these scores are in widespread clinical use [6–9].

In general, tracheostomy is one of the most frequently performed surgical interventions. Although it is a rather short procedure it can be associated with severe complications such as bleeding, wound infection, airway obstruction, pneumonia or tracheal stenosis. The rate of complications ranges from 4% to 40% depending on the study [10–16]. Elective tracheostomy performed in an appropriate controlled setting is associated with a lower complication rate than those performed under emergency conditions [17–20]. By definition, acute unplanned tracheostomies are usually poorly controlled procedures that should be avoided whenever possible.

Therefore our retrospective data analysis aimed to establish risk factors for unplanned tracheostomy during panendoscopy to improve the pre-operative risk evaluation and patient consent.

## Patients and methods

**Fig 1:**
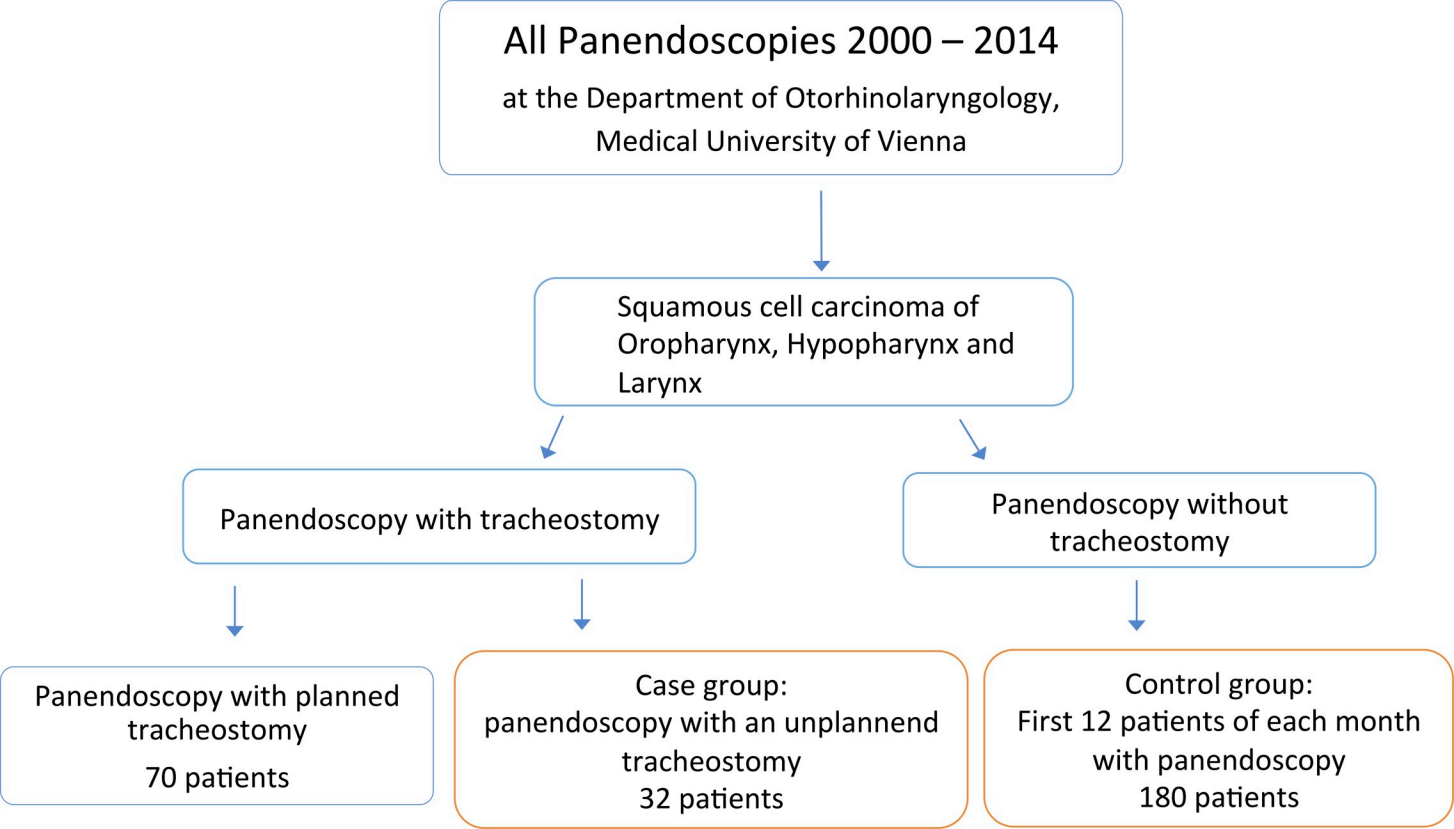
The study design of a retrospective case-control study to assess risk factors for acute unplanned tracheostomy during panendoscopy performed at the Medical University of Vienna between 2000 at 2014. The control group consisted of the first 12 patients of each month undergoing panendoscopy.

In this case-control-study we included all patients undergoing panendoscopy for histologically confirmed HNSCC at the Department of Otolaryngology, Head and Neck Surgery at the Medical University of Vienna between 2000 and 2014. Approval for this retrospective analysis was obtained from the Ethics Committee of the Medical University of Vienna prior to enrolment [1090/2015] and waived the need for consent. Data was accessed anonymously. Patients who required unplanned tracheostomy during panendoscopy were grouped into the case group (n = 32). The control-group included the first consecutive 12 patients of every month that underwent panendoscopy without tracheostomy (n = 180)[Fig 1]. Patients with a present tracheostomy, planned tracheostomy, malignancies other than squamous cell carcinoma or with tumour localisation other than oropharynx, hypopharynx or larynx were excluded from this study. Clinicopathologic data was retrieved from the hospital medical records and evaluated for increased risk for unplanned tracheostomy during panendoscopy. The primary objective was to detect the difference between the tumour localisation (oropharynx, hypopharynx or larynx) and the probability of unplanned tracheostomy during panendoscopy. Possible risk factors included primary or recurrent disease (time of diagnosis), tumour stage, sex, blood clotting disorder, body mass index (BMI), chronic obstructive pulmonary disease (COPD) and history of radiotherapy (RT). Blood clotting disorder was defined as elevated activated partial thromboplastin time (aPTT) measured in the time two weeks before and after the panendoscopy. Patients were staged according to the TNM classification of the Union for International Cancer Control (UICC) [21].

### Statistical analysis

The influence of the binary factors on the risk of unplanned tracheostomy was tested by Fisher`s Exact Test (categorical variables) and T-Test (continuous variables). Univariate and multivariate exact logistical regression models were performed in order to quantify the unadjusted and adjusted effects of the investigated risk factors (tumour localisation, tumour staging and sex. A *p*—level (*p* < 0.05) was used to be statistically significant.

All analyses were calculated using Statistical Analysis Software (SAS, Version 9.4, 2002–2012, SAS Institute Inc., Cary, NC, USA) and Statistical Package for the Social Sciences (SPSS, Version 23.0, SPSS Inc.; Chicago; USA).

## Results

### Patient characteristics

A total of 2131 panendoscopies were performed in patients with HNSCC at the Department of Otolaryngology, Head and Neck Surgery between 2000 and 2014. Patients who underwent planned tracheostomy (n = 70) or with a tumour localisation in nasopharynx (n = 2) were excluded from further analysis. In the case group no patients with oral cancer were found. Subsequently 32 of 2059 patients had an unplanned tracheostomy during panendoscopy, representing 1.6% of all cases. The control group consisted of 180 patients without tracheostomy. The study group hence comprised 212 patients. The descriptive clinical data are presented in Table 1.

**Table 1:** Characteristics of patients undergoing panendoscopy.

Variable		Tracheostomy		
		yes	no	total
		N* (%)	N* (%)	N* (%)
**Sex**	Male	29 (90.6%)	142 (78.9%)	171 (80.7%)
	Female	3 (9.4%)	38 (21.1%)	41 (19.3%)
**Age (range)**		59 (50–94)	60 (40–98)	60 (40–98)
**Tumour**	Oropharynx	6 (18.8%)	93 (51.7%)	99 (46.7%)
**localisation**	Hypopharynx	12 (37.5%)	35 (19.4%)	47 (22.2%)
	Larynx	14 (43.8%)	52 (28.9%)	66 (31.1%)
**Tumour staging**	Early	3 (9.4%)	39 (21.7%)	42 (19.8%)
	Advanced	29 (90.6%)	141 (78.3%)	170 (80.2%)
**Time of**	Primary	24 (75%)	128 (71.1%)	152 (71.7%)
**diagnosis**	Recurrent disease	8 (25%)	52 (28.9%)	60 (28.3%)
**Blood clotting disorder**	Yes	6 (18.8%)	10 (6.2%)	16 (8.3%)
	No	26 (81.3%)	151 (93.8%)	177 (91.7%)
**BMI**^‡^	(in kg/m^2^)	24 (SD^†^ 4.3)	24 (SD^†^ 5.1)	24 (SD^†^ 5)
**COPD**^§^	Yes	14 (43.8%)	52 (28.9%)	66 (31.1%)
	No	18 (56.3%)	128 (71.1%)	146 (68.9%)
**Radiotherapy**	Yes	8 (25%)	47 (26.1%)	55 (25.9%)
	No	24 (75%)	133 (73.9%)	157 (74.1%)
**Timing of**	Intubation	7 (21.9%)		
**tracheostomy**	Extubation	25 (78.1%)		

*Number of patients (%) except stated otherwise.

† standard deviation

Abbreviations

‡ body mass index

§ chronic obstructive pulmonary disease

There were more men (80.7%) than women (19.3%) with a median age of 59.7 years (range 46–93). The tumour was most frequently found in the oropharynx (46.7%), followed by the larynx (31.1%) and hypopharynx (22.2%). An early staging, defined as T1 or T2, negative lymph nodes and absence of distant metastasis, was found in 42 patients (19.8%), whereas the majority of patients (80.2%) had an advanced stage.

In this study, 60 patients (28.3%) had recurrent disease. An extended aPTT was noted in 16 patients (8.3%). In the control group, 19 patients (9%) had no available aPPT on record.

In addition the mean BMI was 24.3 kg/m^2^, COPD was diagnosed in 66 patients (31.1%) and the majority of the patients (74.1%) had not been treated by radiotherapy before panendoscopy. Problems during extubation leading to tracheostomy were found in 25 patients (78.1%) compared to problems with intubation identified in 7 patients (21.9%).

### Risk factors and regression analysis

We found that tumour localisation (*p* = 0.001) and elevated aPTT (*p* = 0.03) but not tumour stage, sex, primary versus recurrent disease, COPD, BMI, age of the patient and radiotherapy influenced the risk for unplanned tracheostomy [Table 2].

**Table 2:** Risk factors correlated with unplanned tracheostomy during panendoscopy.

Variable	*p*-value
Tumour localisation	0.001
Sex	0.149
Tumour staging	0.148
Time of diagnosis	0.832
Blood clotting disorder	0.03
COPD	0.102
Radiotherapy	1.00
Age of the patient	0.597
BMI	0.997

Using univariate regression models [Table 3], only the tumour localisation (*p* = 0.005) but not the tumour stage significantly predicted the need of tracheostomy. Patients with a tumour located in the oropharynx had an almost 7 times lower risk for a tracheostomy than patients with a tumour located in the larynx (OR 0.240; 95% CI 0.087–0.661).

**Table 3:** Univariate and multiple logistic regression models.

	*p*-value	OR	95% CI
**Univariate analysis**			
Tumour localisation	0.005		
Oropharynx vs. larynx		0.240	0.087–0.661
Hypopharynx vs. larynx		1.273	0.527–3.077
Tumour staging	0.120	2.673	0.773–9.242
Sex	0.134	2.587	0.747–8.950
Localisation	0.001		
Oropharynx vs. larynx		0.132	0.045–0.389
Hypopharynx vs. larynx		0.710	0.272–1.854
**Multivariate analysis**			
Staging (advanced vs. early)	0.011	5.697	1.481–21.915
Localisation	0.008		
Oropharynx vs. larynx		0.244	0.088–0.676
Hypopharynx vs. larynx		1.187	0.487–2.892
Sex (male vs. female)	0.242	2.145	0.600–7.663

Multivariate analysis showed that the variables tumour localisation (*p* = 0.001) and tumour stage (*p* = 0.011) were significantly associated with unplanned tracheostomy during panendoscopy [Table 3].

Patients with an advanced tumour had a 5.7 times higher risk of unplanned tracheostomy than patients without an advanced tumour (OR 5.697; 95% CI 1.481–21.915). Comparing oropharynx versus larynx adjusted for the tumour staging, the risk of patients needing a tracheostomy with a tumour located in the oropharynx was 0.13 of those with the tumour located in the larynx (OR 0.132; 95% CI 0.045–0.389). Analysing the variables sex and tumour localisation with the second multiple logistic regression model [Table 3], only the tumour localisation showed a statistical significant influence.

## Discussion

Both, difficult intubation and problems with extubation can lead to unplanned tracheostomies during panendoscopies. An unplanned surgical airway during intubation or extubation is usually the consequence of a “cannot intubate, cannot ventilate” situation—by definition a life threating emergency. As it has to be performed within a few seconds to minutes the procedure is usually not as systematic and safe as a planned tracheostomy. Also tumour patients often present with severe fibrosis, scaring or even pretracheal masses that make the procedure even more difficult. Therefore unplanned tracheostomies should be avoided whenever possible.

In our study unplanned tracheostomy was necessary in 32 out of 2059 patients, corresponding to 1.6% of all cases. This is in line with previous studies where the rate of tracheostomy as a complication of panendoscopy was around 1% [22,23]. Waldron and colleagues investigated the rate of complications during elective and emergency tracheostomies. They found that the presence of a tumour was the most common indication for emergency tracheostomies (60.5%) and that most elective tracheostomies were described in head and neck surgery (72 of 111 patients, 64.3%) [19]. Additionally, several studies found more frequent complications of tracheostomy in emergency conditions illustrating the high risk of these patients [18,24]. Kumar and colleagues described such a case where an unplanned tracheostomy was performed caused by intraoperative findings during panendoscopy and this tracheostomy was associated with post-operative complications like bilateral pulmonary infiltrates and pneumothoraxes [25].

Several scoring systems that asses the risk of tracheostomy in general in head and neck surgery exist but data on patients with HNSCC are sparse [6–9]. Analysing data between 2000 and 2014, our retrospective case-control study investigated the effect of the tumour localisation as a predictor for acute unplanned tracheostomy during panendoscopy. We aimed to detect further risk factors by including age of the patient, sex, tumour staging, time of diagnosis, abnormal aPTT, BMI, COPD and radiotherapy as variables.

Results of our study revealed that there is an association between laryngeal carcinoma and unplanned tracheostomy during panendoscopy. Using a univariate logistic regression model patients with a laryngeal cancer had a 7 times higher risk for tracheostomy than patients with a oropharyngeal cancer. Comparing these two localisations adjusted for staging and multiple testing, patients with an advanced laryngeal tumour showed an almost 6 times higher risk for unplanned tracheostomy than patients with an early stage oropharyngeal carcinoma. This is in line with other published studies. Kruse-Lösler and colleagues also confirmed the tumour localisation as the main predictor for unplanned tracheostomy [6]. They developed a scoring system for the need of elective tracheostomy in patients with oral cavity cancer and identified tumour size, pulmonary diseases, liver diseases, multiple complications, pathologic chest X-rays and regular alcohol intake as further risk factors. In accordance with this study the risk factors recurrent disease and age of the patient did not correlate with unplanned tracheostomy. The other risk factors such as pulmonary disease and multimorbidity identified by Kruse-Lösler and colleagues, were not significant in our study. However, it must be noted that in contrast to our study, they examined oral cancer only. Squamous cell carcinomas located in the larynx or hypopharynx were not evaluated. In a similar study Kim and co-workers also showed a significant association between high tumour stage and high probability of tracheostomy in patients with oral cavity cancer [8].

As yet, no studies have identified clotting disorders as risk factors for tracheostomy. In our study prolonged aPTT was only significant in univariate but not in multivariate analysis. Several studies investigated the effect of the BMI influencing tracheal intubation, complication rate and mortality. In one publication high BMI was associated with higher frequency of difficult tracheal intubation and showed a statistical significance only in the male population [26]. In some studies a higher complication rate was found in obese patients, ranging from 25% to 55%, compared to non-obese patients after tracheostomy [27,28]. Darrat and Yaremchuk found a higher mortality rate in obese patients (BMI 35 kg/m^2^) compared to non-obese patients (28.66% vs. 17.9%) [29].

From clinical experience we would have expected that both, recurrent disease and radiotherapy, influence the risk for tracheotomy. However, these two factors were not significant in our study. We acknowledge the limitations of the retrospective design and the exclusion of tumours found in the oral cavity, nasopharynx and oesophagus in this current study. As expected, the number of patients needing acute tracheostomy was relatively small, which resulted in the fact that the BMI could not be divided into subgroups (normal, obese and morbid BMI). Due to the small numbers of cases we could no evaluated more than three variables by using multivariate regression model. Comparing with other studies we examined the most common risk factors [6,8]. For more detailed analysis future studies should be involve more cases to identify all essential factors influencing tracheostomy.

To our knowledge, this is the first study to identify elevated aPTT as a significant risk factor for unplanned tracheostomy during diagnostic panendoscopy in HNSCC patients. Furthermore we could show that laryngeal cancer and advanced disease were associated with more frequent unplanned tracheostomies. These patients call for a thorough pre-operative evaluation before panendoscopy and a planned tracheostomy should be performed under local anesthesia if there is any doubt about the safety of the airway. However, further prospective studies are needed to further assess risk factors, in order to give clinicians a valid scoring system for preoperative evaluation.
